# Association of the stress hyperglycemia ratio with coronary artery disease complexity as assessed by the SYNTAX score in patients with acute coronary syndrome

**DOI:** 10.1186/s13098-024-01382-0

**Published:** 2024-06-25

**Authors:** Sheng Zhao, Zuoxiang Wang, Ping Qing, Minghui Li, Qingrong Liu, Keke Wang, Xiaojin Gao, Jie Zhao, Yongjian Wu

**Affiliations:** 1 Fuwai Hospital, National Centre for Cardiovascular Diseases, National Clinical Research Centre for Cardiovascular Diseases, Chinese Academy of Medical Sciences and Peking Union Medical College, Beijing, China; 2 Department of Cardiology, Beijing Aerospace General Hospital, Beijing, China; 3 Department of Cardiology, the Second Medical Centre, Chinese PLA General Hospital, Beijing, China; 4 Fuwai Hospital, National Centre for Cardiovascular Diseases, National Clinical Research Centre for Cardiovascular Diseases, Chinese Academy of Medical Sciences and Peking Union Medical College, No.167 North Lishi Road, Xicheng District, Beijing, 100037 China

**Keywords:** Stress hyperglycemia ratio, Coronary artery disease, Acute coronary syndrome, Diabetes mellitus

## Abstract

**Background:**

Mounting evidence supports a significant correlation between the stress hyperglycemia ratio (SHR) and both short- and long-term prognoses in patients with acute coronary syndrome (ACS). Nevertheless, research examining the association between the SHR and the complexity of coronary artery disease (CAD) is scarce. Therefore, this study aimed to explore the association between the SHR and CAD complexity, as assessed by the SYNTAX score, in patients with ACS.

**Methods:**

A total of 4715 patients diagnosed with ACS were enrolled and divided into five groups according to the quintiles of the SHR. CAD complexity was assessed using the SYNTAX score and categorized as low (≤ 22) or mid/high (> 22) levels. Logistic regression was utilized to examine the association between the SHR and CAD severity (mid-/high SYNTAX score). Restricted cubic spline (RCS) curves were generated to assess the association between the SHR and CAD severity. Subgroup analyses were conducted to stratify outcomes based on age, sex, diabetes mellitus (DM) status, and clinical presentation.

**Results:**

Among the total ACS population, 503 (10.7%) patients had mid/high SYNTAX scores. Logistic regression analysis revealed that the SHR was an independent risk factor for mid/high SYNTAX scores in a U-shaped pattern. After adjusting for confounding variables, Q1 and Q5 demonstrated elevated odds ratios (ORs) relative to the reference category Q3, with ORs of 1.61 (95% CI: 1.19 ∼ 2.19) and 1.68 (95% CI: 1.24 ∼ 2.29), respectively. Moreover, the ORs for Q2 (1.02, 95% CI: 0.73 ∼ 1.42) and Q4 (1.18, 95% CI: 0.85 ∼ 1.63) resembled that of Q3. Compared with the merged Q2-4 group, the ORs were 1.52 (95% CI: 1.21 ∼ 1.92) for Q1 group and 1.58 (95% CI: 1.25 ∼ 2) for the Q5 group. Subgroup analysis revealed that the U-shaped association between the SHR and mid/high SYNTAX score was attenuated in DM patients (P for interaction = 0.045).

**Conclusions:**

There were U-shaped associations between the SHR and CAD complexity in ACS patients, with an SHR ranging from 0.68 to 0.875 indicating a relatively lower OR for mid/high SYNTAX scores. Further studies are necessary to both evaluate the predictive value of the SHR in ACS patients and explore the underlying mechanisms of the observed U-shaped associations.

**Supplementary Information:**

The online version contains supplementary material available at 10.1186/s13098-024-01382-0.

## Background

The complexity of coronary artery disease (CAD) directly correlates with its severity and is linked to adverse clinical outcomes [1, 2]. The Synergy between the PCI with TAXUS™ and Cardiac Surgery (SYNTAX) score is a widely used angiographic tool for grading the complexity of CAD and helps in decision-making between coronary artery bypass grafting surgery (CABG) and percutaneous coronary intervention (PCI) for patients with complex CAD [1, 3–6]. Patients with intermediate to high SYNTAX scores (> 22) face a heightened risk of major adverse cardiovascular events (MACEs) and are better candidates for CABG [1, 4, 5, 7, 8]. Diabetes and blood glucose levels are closely linked to CAD development as traditional risk factors. However, the direct correlation between acute and chronic blood glucose markers and the SYNTAX score remains debating [6, 9–15].

The stress hyperglycemia ratio (SHR) is a novel marker intended to reflect genuine glucose status and estimate relative hyperglycemia [16, 17]. Increasing evidence supports a significant association between the SHR and short- and long-term adverse cardiovascular outcomes in patients with acute coronary syndrome (ACS) [18, 19]. A recent study investigated the association between this ratio and CAD severity, revealing a noteworthy correlation between the SHR and the incidence of multivessel CAD (MVD) [20]. However, there is currently no research examining the relationship between the SHR and CAD complexity in ACS patients using the more comprehensive SYNTAX score as an indicator of CAD complexity.

Therefore, the aim of this study was to investigate the complexity of CAD across the SHR continuum and assess the association between the SHR and the SYNTAX score in patients with ACS.

## Methods

### Study design and population

**Fig. 1:**
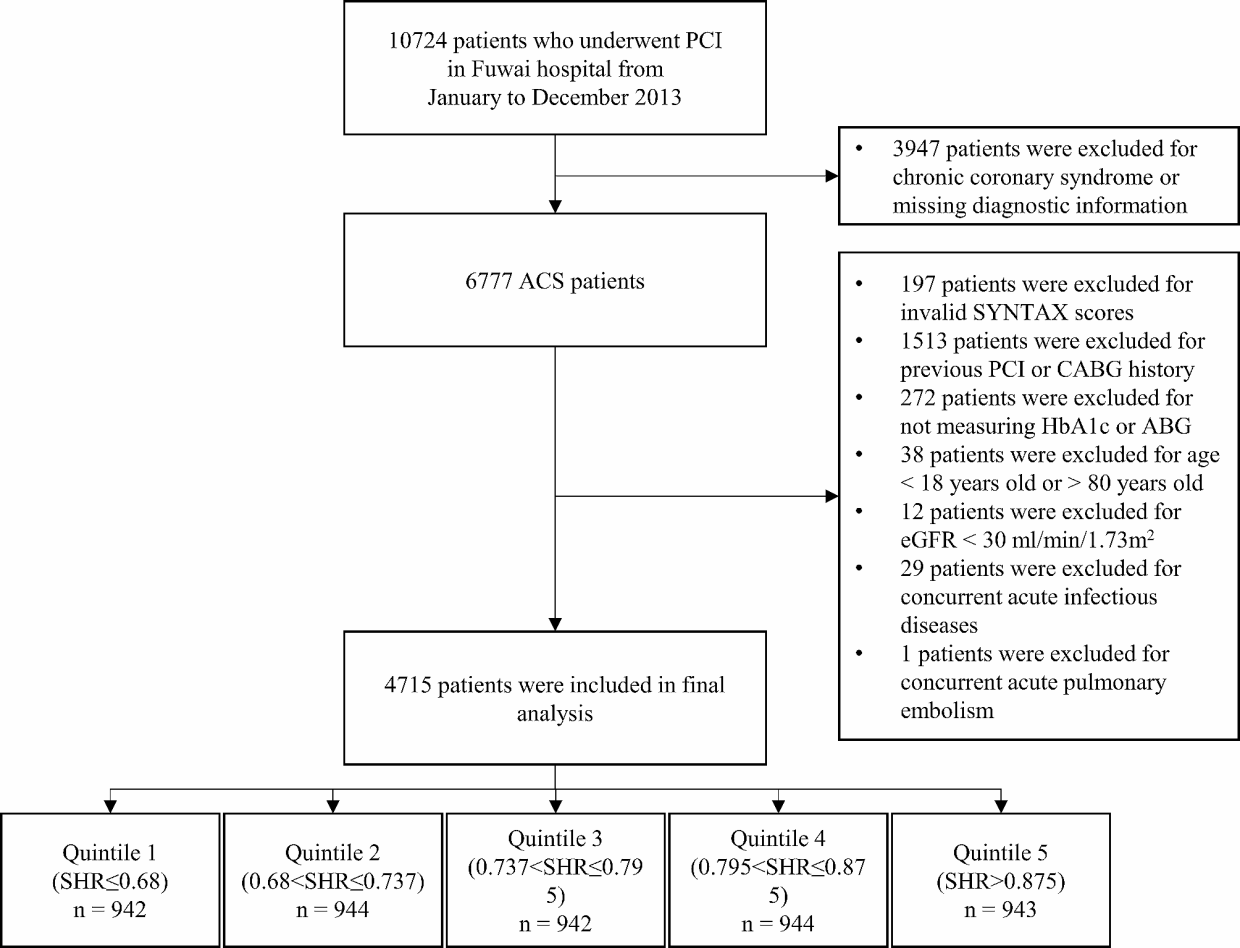
Flowchart

This study was a prospective, observational cohort study conducted at Fuwai Hospital, National Center for Cardiovascular Diseases. The study adhered to the principles outlined in the Declaration of Helsinki and received approval from the Fuwai Hospital Ethics Review Committee. All participants provided informed consent prior to enrollment. From January 1, 2013, to December 31, 2013, a total of 10,724 patients who underwent percutaneous coronary intervention (PCI) at Fuwai Hospital were consecutively screened. The inclusion criterion was patients who presented with acute coronary syndrome (ACS). The following exclusion criteria were used: (1) had an invalid SYNTAX score, (2) had a previous PCI or CABG history, (3) lacked crucial laboratory data (admission glucose or glycated hemoglobin A1c [HbA1c]), (4) were < 18 years old or > 80 years old, (5) had an eGFR < 30 ml/min/1.73 ^m2^, and (6) had other cooccurring diseases in the acute phase. Ultimately, 4715 ACS patients were successfully enrolled and divided into five groups based on quintiles. The detailed process of population enrollment is depicted in Fig. 1.

**Fig. 2:**
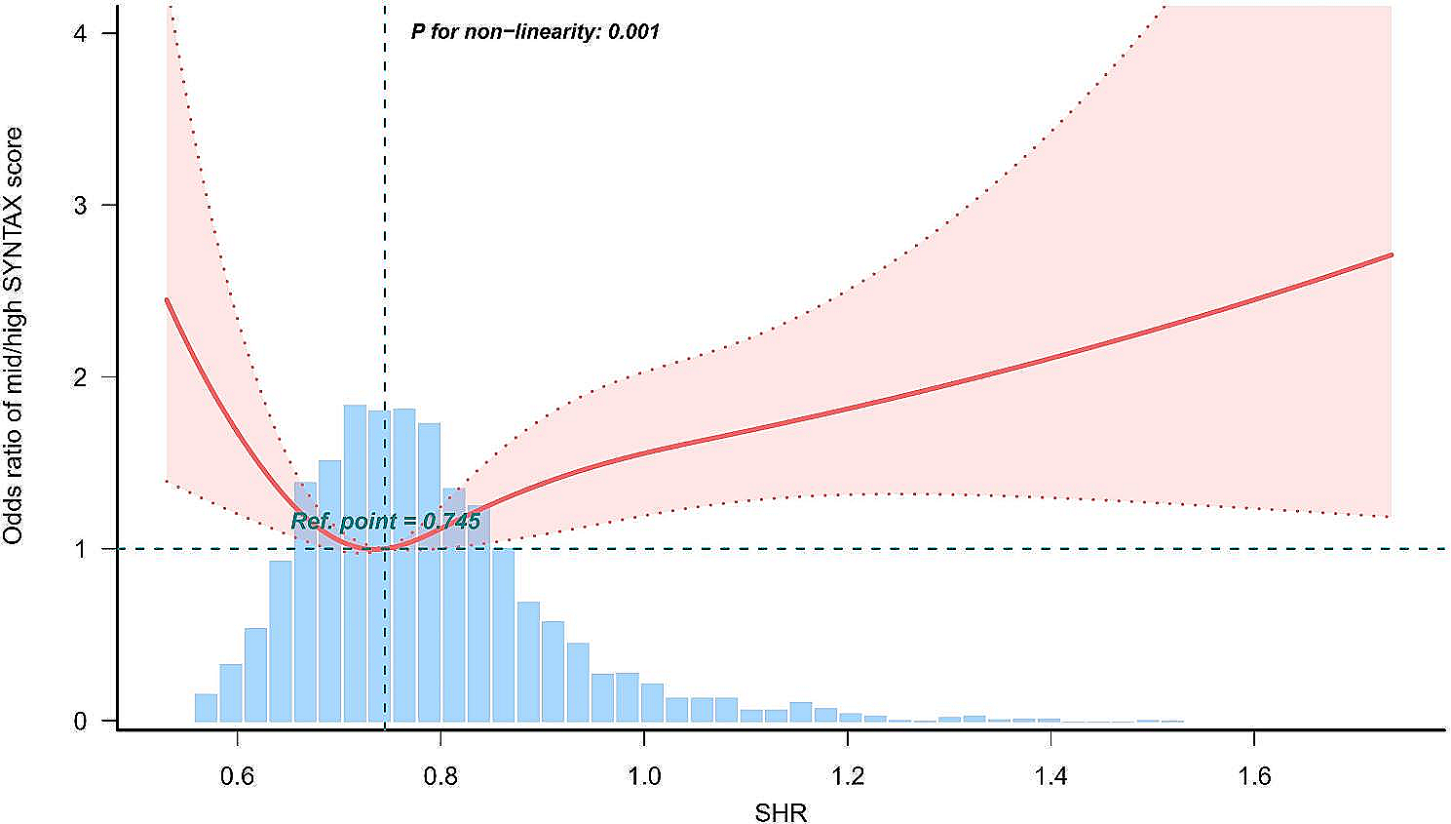
Restricted cubic splines for the odds ratio of mid/high SYNTAX score. Adjusted for age, sex, body mass index, current smoker, DM, hypertension, dyslipidemia, previous MI, previous stroke, PVD, eGFR, TG, HDL-C, LDL-C, Lp (a), hs-CRP, uric acid, LVEF < 40% and clinical presentation. Abbreviations as shown in Table 1

**Table 1:** Baseline patient characteristics according to SHR levels

Variable	Total *n* = 4715	Q1 (SHR ≤ 0.68) *n* = 942	Q2 (0.68 < SHR ≤ 0.737) *n* = 944	Q3 (0.737 < SHR ≤ 0.795) *n* = 942	Q4 (0.795 < SHR ≤ 0.875) *n* = 944	Q5 (SHR > 0.875) *n* = 943	*P* value
SHR	0.79 ± 0.16	0.62 ± 0.06	0.71 ± 0.02	0.77 ± 0.02	0.83 ± 0.02	1.03 ± 0.18	< 0.001
Age, year	57.88 ± 10.26	58.93 ± 10.53	58.13 ± 10.30	57.82 ± 10.01	57.36 ± 10.12	57.17 ± 10.27	0.002
Male, n (%)	3542 (75.1)	678 (72)	694 (73.5)	708 (75.2)	706 (74.8)	756 (80.2)	< 0.001
BMI, kg/m^2^	25.83 ± 3.28	25.52 ± 3.49	25.96 ± 3.14	25.75 ± 3.30	25.92 ± 3.20	26.01 ± 3.22	0.006
SBP, mmHg	126.75 ± 16.65	126.02 ± 17.00	126.24 ± 16.62	126.69 ± 16.40	126.57 ± 16.56	128.21 ± 16.59	0.038
DBP, mmHg	77.49 ± 10.63	76.69 ± 10.26	77.58 ± 10.71	78.12 ± 11.07	77.56 ± 10.49	77.49 ± 10.58	0.070
Current smoker, n (%)	2722 (57.7)	526 (55.8)	549 (58.2)	556 (59)	532 (56.4)	559 (59.3)	0.44
DM, n (%)	1909 (40.5)	498 (52.9)	293 (31)	303 (32.2)	319 (33.8)	496 (52.6)	< 0.001
Hypertension, n (%)	2997 (63.6)	602 (63.9)	600 (63.6)	576 (61.1)	609 (64.5)	610 (64.7)	0.508
Dyslipidemia, n (%)	3017 (64.0)	621 (65.9)	609 (64.5)	588 (62.4)	617 (65.4)	582 (61.7)	0.237
Previous MI, n (%)	1671 (35.4)	338 (35.9)	319 (33.8)	304 (32.3)	294 (31.1)	416 (44.1)	< 0.001
Previous stroke, n (%)	523 (11.1)	121 (12.8)	126 (13.3)	78 (8.3)	105 (11.1)	93 (9.9)	0.002
PVD, n (%)	258 (5.5)	57 (6.1)	57 (6)	41 (4.4)	49 (5.2)	54 (5.7)	0.442
COPD, n (%)	112 (2.4)	25 (2.7)	24 (2.5)	24 (2.5)	14 (1.5)	25 (2.7)	0.393
eGFR, mL/min/1.73m^2^	91.79 ± 14.75	91.28 ± 14.44	91.77 ± 14.84	92.09 ± 14.15	93.28 ± 14.04	90.55 ± 16.08	0.001
LVEF, %	63.16 ± 6.86	63.36 ± 6.83	63.60 ± 6.60	63.51 ± 6.60	63.45 ± 6.56	61.88 ± 7.53	< 0.001
LVEF < 40%, n (%)	38 (0.8)	7 (0.7)	5 (0.5)	7 (0.7)	6 (0.6)	13 (1.4)	0.267
Clinical presentation							< 0.001
UA, n (%)	3267 (69.3)	667 (70.8)	678 (71.8)	686 (72.8)	682 (72.2)	554 (58.7)	
NSTEMI, n (%)	418 (8.9%)	70 (7.4)	88 (9.3)	71 (7.5)	80 (8.5)	109 (11.6)	
STEMI, n (%)	1030 (21.8%)	205 (21.8)	178 (18.9)	185 (19.6)	182 (19.3)	280 (29.7)	
Laboratory data							
TG, mmol/L	1.80 ± 1.04	1.76 ± 1.05	1.73 ± 0.90	1.79 ± 0.97	1.76 ± 0.96	1.93 ± 1.29	< 0.001
TC, mmol/L	4.26 ± 1.05	4.13 ± 1.00	4.16 ± 1.06	4.28 ± 1.02	4.30 ± 1.05	4.44 ± 1.09	< 0.001
HDL-C, mmol/L	1.03 ± 0.28	1.00 ± 0.27	1.02 ± 0.27	1.03 ± 0.28	1.05 ± 0.29	1.04 ± 0.28	0.003
LDL-C, mmol/L	2.55 ± 0.90	2.45 ± 0.83	2.49 ± 0.92	2.56 ± 0.87	2.59 ± 0.91	2.69 ± 0.94	< 0.001
Lp (a), mg/L	288.62 ± 280.23	299.34 ± 273.11	299.89 ± 290.43	282.96 ± 276.89	273.57 ± 277.85	287.35 ± 282.21	0.197
Hs-CRP, mg/L	3.62 ± 4.06	3.30 ± 3.66	3.41 ± 3.79	3.30 ± 3.85	3.33 ± 3.83	4.77 ± 4.85	< 0.001
ALB, g/L	42.63 ± 4.22	41.19 ± 3.81	42.20 ± 3.76	43.02 ± 4.09	43.62 ± 4.21	43.14 ± 4.71	< 0.001
ABG, mmol/L	6.23 ± 2.16	5.24 ± 1.19	5.43 ± 1.19	5.78 ± 1.24	6.22 ± 1.43	8.49 ± 3.17	< 0.001
HBA1c, %	6.58 ± 1.25	7.00 ± 1.41	6.43 ± 1.05	6.37 ± 1.01	6.34 ± 1.06	6.76 ± 1.50	< 0.001
Hemoglobin, %	142.58 ± 15.43	139.61 ± 15.36	141.14 ± 14.76	143.58 ± 15.27	144.24 ± 14.88	144.33 ± 16.31	< 0.001
Uric acid, mmol/L	340.06 ± 84.88	341.17 ± 84.85	345.57 ± 85.18	342.34 ± 81.94	339.26 ± 83.29	331.96 ± 88.59	0.009
Angiographic characteristics							
LM disease, n (%)	238 (5.0)	53 (5.6)	57 (6)	46 (4.9)	35 (3.7)	47 (5)	0.186
TVD, n (%)	1810 (38.4)	370 (39.3)	352 (37.3)	376 (39.9)	332 (35.2)	380 (40.3)	0.12
Long lesion, n (%)	2730 (57.9)	544 (57.7)	539 (57.1)	534 (56.7)	539 (57.1)	574 (60.9)	0.344
Calcified lesions, n (%)	686 (14.5)	142 (15.1)	137 (14.5)	119 (12.6)	125 (13.2)	163 (17.3)	0.041
Thrombosis, n (%)	153 (3.2)	21 (2.2)	27 (2.9)	16 (1.7)	26 (2.8)	63 (6.7)	< 0.001
CTO disease, n (%)	723 (15.6)	144 (15.7)	128 (13.7)	120 (13)	145 (15.7)	186 (20)	< 0.001
SYNTAX score	11.81 ± 7.73	12.41 ± 8.26	11.61 ± 7.36	11.43 ± 7.25	11.12 ± 7.39	12.47 ± 8.26	< 0.001
SYNTAX score > 22	503 (10.7)	126 (13.4)	80 (8.5)	76 (8.1)	87 (9.2)	134 (14.2)	< 0.001

Abbreviations: ABG: admission blood glucose; ALB: albumin; BMI: body mass index; COPD: chronic obstructive pulmonary disease; CTO: chronic total occlusion; DBP: diastolic blood pressure; eGFR: estimated glomerular filtration rate; Hs-CRP: high-sensitivity C-reactive protein; HDL-C: high density lipoprotein; LDL-C: low density lipoprotein; LM: left main disease; LVEF: left ventricular ejection fraction; MI: myocardial infarction; MVD: multivessel disease; NSTEMI: non-ST-segment elevation myocardial infarction; PVD: peripheral vascular disease; SBP: systolic blood pressure; STEMI: ST-segment elevation myocardial infarction; TG: triglyceride; TC: total cholesterol; UA: unstable angina; SYNTAX: Synergy between PCI with TAXUS™ and Cardiac Surgery. Data are presented as the mean ± SD; median (IQR) or n (%)

### Data collection and definitions

We collected baseline demographic and clinical data prospectively for all patients. Demographic information included age, sex, BMI, comorbidities, smoking status, and prior history of myocardial infarction (MI). Clinical data included clinical presentation, laboratory test results, auxiliary examination findings and angiographic characteristics. Glycemic status upon admission was assessed using the LABOSPECT 008 system (Hitachi, Tokyo, Japan), and the glycated hemoglobin (HbA1c) level was determined using high-performance liquid chromatography (G8; TOSOH, Tokyo, Japan). Body mass index (BMI) was determined by dividing weight (in kilograms) by the square of height (in meters). The estimated glomerular filtration rate (eGFR) was assessed using the Chronic Kidney Disease Epidemiology Collaboration creatinine equation [21]. Following the completion of coronary angiography (CAG), the characteristics of coronary disease, including the number of stenotic vessels, unusual types of coronary stenosis, and SYNTAX score, were assessed by two coronary intervention specialists [3].

The SHR was calculated using the following formula: ABG (mmol/l)/[1.59×HbA1c (%) − 2.59] [16, 17]. Acute coronary syndrome (ACS) was defined as unstable angina (UA), non-ST segment elevation myocardial infarction (NSTEMI), or ST segment elevation myocardial infarction (STEMI) [22]. Diabetes status was documented for patients with a prior diagnosis of diabetes, current or previous use of oral hypoglycemic drugs or insulin, or HbA1c levels > 6.5%. CAD was defined as the presence of at least one major coronary artery with ≥ 50% stenosis confirmed by CAG, including the left anterior descending, left circumflex, and right coronary arteries. The severity of CAD was assessed using the SYNTAX score, categorizing patients into two groups based on their scores: the low SYNTAX score group with scores ≤ 22 and the mid/high SYNTAX score group with scores > 22. Participants with one major coronary artery with ≥ 50% stenosis were categorized as having single-vessel CAD, while those with stenosis in more than two coronary arteries were classified as having multivessel CAD. Additionally, left main (LM) disease was defined as > 50% stenosis in the left main coronary artery, which also qualifies as multivessel CAD.

### Statistical analysis

Continuous variables are expressed as the mean ± standard deviation (SD) or as the median and interquartile range (IQR), while categorical variables are presented as numbers and percentages. Statistical analyses included the χ2 test for comparing categorical variables and the t test, analysis of variance, Mann–Whitney U test, or Kruskal–Wallis H test for continuous variables. Logistic regression was utilized to examine the association between the SHR and CAD severity (mid/high SYNTAX score), with odds ratios (ORs) and corresponding 95% confidence intervals (CIs) calculated. Restricted cubic spline (RCS) curves were generated to assess the association between the SHR and CAD severity. Furthermore, subgroup analyses were conducted to stratify outcomes based on age, sex, diabetes mellitus (DM), and clinical presentation. These analyses were performed using comprehensive regression models adjusted for potential confounders. All the statistical analyses were performed using R version 4.3.0 software (R Foundation for Statistical Computing, Vienna, Austria), with statistical significance set at a *P* value < 0.05.

## Results

### Baseline characteristics

A total of 4715 patients with ACS were included in our study. The mean age was 57.88 ± 10.26 years, with 3542 (75.1%) being male. The mean body mass index (BMI) was 25.83 ± 3.28 kg/m^2^. Among the enrolled patients, 3267 (69.3%) presented with unstable angina (UA), while 1418 (30.7%) were diagnosed with acute myocardial infarction (AMI). Hypertension was prevalent in 2997 (63.6%) patients, and hyperlipidemia was diagnosed in 3017 (64.0%) patients. Additionally, 1909 (40.5%) patients had diabetes, and 57.7% were current smokers. The mean SYNTAX score was 11.81 ± 7.73, with 10.7% of patients having a mid/high SYNTAX score (> 22). Patients were stratified into five groups based on the SHR: the Q1 group (SHR ≤ 0.68), *n* = 942; the Q2 group (0.68 < SHR ≤ 0.737), *n* = 944; the Q3 group (0.737 < SHR ≤ 0.795), *n* = 942; the Q4 group (0.795 < SHR ≤ 0.875), *n* = 944; and the Q5 group (SHR > 0.875), *n* = 943.

Significant differences were observed across different SHR groups concerning several demographic and clinical parameters. These included age, sex, BMI, systolic blood pressure (SBP), incidence of diabetes mellitus (DM), and history of previous myocardial infarction (MI) and stroke. Furthermore, noteworthy variations were evident in laboratory parameters, including triglyceride (TG), total cholesterol (TC), high-density lipoprotein cholesterol (HDL-C), low-density lipoprotein cholesterol (LDL-C), lipoprotein (a) [Lp(a)], high-sensitivity C-reactive protein (Hs-CRP), albumin (ALB), admission blood glucose (ABG), glycated hemoglobin (HbA1c), hemoglobin levels, and uric acid levels. Additionally, angiographic characteristics such as left main (LM) disease, triple-vessel disease (TVD), chronic total occlusion (CTO) disease, and SYNTAX score were significantly different across SHRs. Across Q1 to Q5, both age and the proportion of females decreased gradually. Q5 demonstrated a notably greater incidence of previous MI than did the other quintiles. Conversely, the prevalence of DM in Q2, Q3, and Q4 was lower than that in Q1 and Q5. The incidence of AMI increased progressively from Q1 to Q5, while that of UA decreased. Laboratory parameters such as TG, TC, HDL-C, LDL-C, hs-CRP, ALB, and hemoglobin steadily increased from Q1 to Q5. ABG and HBA1c showed contrasting trends, with ABG increasing and HBA1c decreasing. For most angiographic characteristics, the proportion of complex lesions in Q2, Q3, and Q4 was lower than that in Q1 and Q5, resulting in similar distribution trends observed for the SYNTAX scores. The detailed baseline characteristics are presented in Table 1.

### Associations between the SHR and CAD complexity assessed by the SYNTAX score

Among the total ACS population, 503 (10.7%) patients had mid/high SYNTAX scores (> 22). Logistic regression analyses revealed a significant U-shaped association between the SHR and mid/high SYNTAX score in ACS patients, as shown in Table 2. According to the unadjusted model, Q1 and Q5 exhibited greater odds ratios (ORs) than Q3 (reference), with ORs of 1.76 (95% CI: 1.3 ∼ 2.38) and 1.86 (95% CI: 1.4 ∼ 2.54), respectively. Moreover, the ORs of Q2 (1.06, 95% CI: 0.76 ∼ 1.46) and Q4 (1.16, 95% CI: 0.84 ∼ 1.6) were similar to those of Q3, with no significant differences observed. This U-shaped association persists consistently in both Model 1 (the basic model) and Model 2 (the comprehensive model). Patients with intermediate SHR levels (Q2, Q3 and Q4) exhibited the lowest odds of risk, with the risk increasing progressively in other groups. After adjusting for all confounding demographic and clinical variables, the ORs for Q1 and Q5 compared to Q3 were 1.61 (95% CI: 1.19 ∼ 2.19) and 1.68 (95% CI: 1.24 ∼ 2.29), respectively. Additionally, no significant differences were detected among Q2, Q3, and Q4. Given the relatively consistent low ORs for mid/high SYNTAX scores in the Q2, Q3, and Q4 groups, we merged them into one group (Q2-4) and reanalyzed them compared to Q1 and Q5. Similar risk associations were identified. Compared to Q2-4, the ORs were 1.52 (95% CI: 1.2 ∼ 1.92) for the Q1 group and 1.58 (95% CI: 1.25 ∼ 2) for the Q5 group.

**Table 2:** Associations between the SHR and mid/high SYNTAX score

		Unadjusted	Model 1	Model 2
	Total/event (%)	OR (95% CI)	OR (95% CI)	OR (95% CI)
**Classification 1**				
Q1	942/126 (13.4)	1.76 (1.3 ∼ 2.38)	1.73 (1.28 ∼ 2.34)	1.61 (1.19 ∼ 2.19)
Q2	944/80 (8.5)	1.06 (0.76 ∼ 1.46)	1.05 (0.76 ∼ 1.46)	1.02 (0.73 ∼ 1.42)
Q3	942/76 (8.1)	1 (reference)	1 (reference)	1 (reference)
Q4	944/87 (9.2)	1.16 (0.84 ∼ 1.6)	1.17 (0.85 ∼ 1.61)	1.18 (0.85 ∼ 1.63)
Q5	943/134 (14.2)	1.89 (1.4 ∼ 2.54)	1.9 (1.41 ∼ 2.56)	1.68 (1.24 ∼ 2.29)
**Classification 2**				
Q1	942/126 (13.4)	1.64 (1.31 ∼ 2.07)	1.61 (1.28 ∼ 2.03)	1.52 (1.2 ∼ 1.92)
Q2-4	2830/243 (8.6)	1 (reference)	1 (reference)	1 (reference)
Q5	943/134 (14.2)	1.76 (1.41 ∼ 2.21)	1.77 (1.41 ∼ 2.22)	1.58 (1.25 ∼ 2)

Model 1: adjusted for age, sex; Model 2: adjusted for age, sex, BMI, current smoker, DM, hypertension, dyslipidemia, previous MI, previous stroke, PVD, eGFR, TG, HDL-C, LDL-C, Lp (a), hs-CRP, uric acid, LVEF < 40% and clinical presentation. Abbreviations as shown in Table 1

To further explore the association between the SHR and CAD complexity, we conducted an RCS analysis, as depicted in Fig. 2. In line with the aforementioned results, the RCS analysis validated a U-shaped pattern in the relationship between the SHR and mid/high SYNTAX scores. The lowest odds ratio (OR) was identified at a nadir SHR of 0.74, corresponding to the 0.737 ∼ 0.795 interval (Q3, reference group).

### Subgroup analysis of the association between the SHR and CAD complexity

Table 3 displays the findings of a stratified analysis examining the association between the SHR and CAD complexity, considering factors such as age, sex, BMI, DM, and clinical presentation. This U-shaped association was consistently observed in various subgroups. This distinct pattern underscores the critical importance of the nadir interval at 0.68 ∼ 0.875, wherein any deviation from this optimal SHR value range (whether increased or decreased) is consistently correlated with an elevated risk of a mid/high SYNTAX score. Significant interactions between SHR and DM (P for interaction = 0.045) were identified. Among patients without DM, the U-shaped association between the SHR and mid/high SYNTAX score was stronger than that between the SHR and DM. This difference was primarily evident in the lowest SHR (Q1), where the OR for Q1 in the non-DM group significantly increased to 2.07 (95% CI: 1.51–2.84) compared to that in the Q2-4 group, while the OR in the DM group showed only an increasing trend to 1.17 (95% CI: 0.84–1.64). At the highest SHR (Q5), there were consistently elevated ORs compared to those in Q2-4, both in the non-DM group and in the DM group, with values of 1.69 (95% CI: 1.21–2.35) and 1.59 (95% CI: 1.15–2.18), respectively.

**Table 3:** Associations between the SHR and mid/high SYNTAX score across different subgroups

Subgroups		SHR levels			*P* value for interaction
	Total/event (%)	Q1 (SHR ≤ 0.68)	Q2-4 (0.68 < SHR ≤ 0.875)	Q5 (SHR > 0.875)	
Age					0.987
< 65 years	3452/330 (9.6)	1.48 (1.1 ∼ 1.98)	1 (reference)	1.58 (1.19 ∼ 2.11)	
≥ 65 years	1263/173 (13.7)	1.56 (1.04 ∼ 2.32)	1 (reference)	1.57 (1.02 ∼ 2.43)	
Sex					0.732
Male	3542/380 (10.7)	1.48 (1.12 ∼ 1.96)	1 (reference)	1.51 (1.15 ∼ 1.97)	
Female	1173/123 (10.5)	1.52 (0.96 ∼ 2.41)	1 (reference)	1.82 (1.08 ∼ 3.08)	
BMI					0.068
< 25 kg/m2	1945/220 (11.3)	1.11 (0.77 ∼ 1.61)	1 (reference)	1.52 (1.05 ∼ 2.19)	
≥ 25 kg/m2	2770/283 (10.2)	1.97 (1.44 ∼ 2.7)	1 (reference)	1.69 (1.23 ∼ 2.31)	
DM					0.045
No	2806/262 (9.3)	2.07 (1.51 ∼ 2.84)	1 (reference)	1.69 (1.21 ∼ 2.35)	
Yes	1909/241 (12.6)	1.17 (0.84 ∼ 1.64)	1 (reference)	1.59 (1.15 ∼ 2.18)	
Clinical presentation					0.451
STEMI	1030/129 (12.5)	1.39 (0.82 ∼ 2.34)	1 (reference)	2.13 (1.34 ∼ 3.38)	
NSTEMI	418/60 (14.4)	1.49 (0.68 ∼ 3.27)	1 (reference)	1.41 (0.69 ∼ 2.91)	
UA	3267/314 (9.6)	1.57 (1.18 ∼ 2.09)	1 (reference)	1.42 (1.04 ∼ 1.95)	

Adjusted for age sex, body mass index, current smoker, DM, hypertension, dyslipidemia, previous MI, previous stroke, PVD, eGFR, TG, HDL-C, LDL-C, Lp (a), hs-CRP, uric acid, LVEF < 40% and clinical presentation. Abbreviations as shown in Table 1

## Discussion

We investigated the correlation between the SHR and CAD complexity in ACS patients. Two main findings were identified. First, the SHR was independently associated with mid/high SYNTAX scores in ACS patients. Second, there was a U-shaped pattern in the associations, with ORs for mid/high SYNTAX scores significantly increasing when the SHR exceeded 0.875 or fell below 0.68.

CAD complexity directly reflects CAD severity, with extensive and intricate CAD consistently linked to adverse clinical outcomes in various studies [1, 2]. Several assessment methods and scoring tools have emerged to evaluate CAD complexity. Among them, the SYNTAX score stands out as a comprehensive tool that integrates multiple scoring systems to evaluate both the extent and anatomical complexity of CAD [3]. Its value lies in two aspects. First, in predicting CAD outcomes, the BARI-2D trial showed that a mid/high SYNTAX score (≥ 23) predicted higher rates of major cardiovascular events (MACEs) [1]. Second, it guides treatment decisions: several studies have indicated that higher SYNTAX scores predict better outcomes with CABG than with PCI [23–25]. Current clinical guidelines for coronary artery revascularization advocate employing the SYNTAX score for evaluating CAD complexity and directing optimal revascularization strategies [4, 5]. In essence, investigating the risk factors correlated with the SYNTAX score is highly important for both mechanistic research and the clinical diagnosis and treatment of CAD. However, current research in this area predominantly focuses on stable CAD, with relatively limited studies conducted in the ACS population.

The SHR is a metric that evaluates the ratio of stress-induced hyperglycemia to chronic glycemia using a specific formula, offering insights into the severity of critical illness [16, 17]. Extensive literature suggests that stress hyperglycemia, caused by neurohormonal dysregulation and inflammatory responses, is common in acutely critically ill patients and acts as a marker of disease severity, including in ACS patients [26, 27]. Stress hyperglycemia reflects ACS severity and may worsen acute cardiac pathology through various mechanisms, including inflammation activation, microcirculatory obstruction, and platelet dysfunction [27–29], all of which are involved in ACS progression and development based on CAD pathology and are also implicated in the progression and development of ACS based on CAD pathology [30, 31]. In addition, as a traditional risk factor for CAD, chronic hyperglycemia can adversely impact inflammation, cellular injury, apoptosis, ischemic myocardial metabolism, endothelial function, the coagulation cascade, and platelet aggregation [32]. As CAD progresses is a chronic condition, HbA1c serves as a sensitive indicator of chronic dysglycemia and is strongly correlated with the SYNTAX score [13, 33, 34]. In summary, the interplay between ACS and acute and chronic hyperglycemia is complex. The integration of HbA1c and acute blood glucose levels by the SHR provides a more accurate assessment of patients’ true blood glucose status, offering a novel research perspective on CAD complexity in ACS patients [16, 17]. Currently, there is limited literature on the relationship between the SHR and CAD complexity. Zhang Y et al. addressed this gap by assessing CAD complexity using single-vessel (SVD) and multivessel disease (MVD) [20]. This study retrospectively enrolled 987 patients who underwent CAG and revealed that the SHR was significantly correlated with the risk of MVD. Logistic regression analysis revealed that the risk of MVD was 1.939-fold greater in the T2 group and 1.860-fold greater in the T3 group than in the T1 group. Regrettably, the study had a relatively small sample size and did not explicitly specify whether the population under investigation consisted of individuals with ACS. Furthermore, it is noteworthy that within the MVD group, considerable heterogeneity in CAD complexity still existed [3]. Our study revealed an independent link between the SHR and complex coronary anatomical lesions evaluated by the SYNTAX score in ACS patients, representing a novel finding in a large-scale cohort.

To the best of our knowledge, this study may be the first to demonstrate a U-shaped correlation between the SHR and mid/high SYNTAX score in ACS patients. We consecutively enrolled ACS patients from a large prospective cohort in Asia. Our findings indicated significantly greater ORs of mid/high SYNTAX scores in the lowest and highest SHR quintiles than in the median quintile. Subsequent RCS analysis revealed a U-shaped correlation with all confounding factors adjusted. Notably, the ORs for mid/high SYNTAX scores remained relatively low in the middle three quintiles (Q2, Q3 and Q4), with no statistically significant differences between them, forming the bottom of the U-shape. The specific mechanism underlying this U-shaped association remains unclear. Indeed, there is growing evidence of this analogous U-shaped phenomenon in both short-term and long-term prognostic studies of ACS, as well as in heart failure and other critical illnesses, implying a widespread association between excessively high or low SHR and adverse effects on the human body [19, 35–39]. Yang J et al. identified U-shaped associations between the SHR and the rates of major adverse cardiac and cerebrovascular events (MACCEs) as well as major adverse cardiac events (MACEs) at the 2-year follow-up in ACS patients treated with DES implantation, with the inflection point determined to be 0.78 [40]. The mechanism underlying this phenomenon may involve moderate stress-induced hyperglycemia, which is a normal physiological response and a protective mechanism during ACS [41]. Both the SYNTAX score and the SHR are related to ACS prognosis, and they may share certain causal mechanisms, thus exhibiting similar effects [1, 8]. Our study suggested that a range of 0.68 to 0.875 may indicate a “moderate stress level”, as levels above or below this range correlate with increased CAD severity. Therefore, an SHR > 0.875 may truly indicate stress-induced hyperglycemia. This condition, coupled with higher CAD complexity, leads to more severe ACS conditions, as evidenced by the significantly increased proportion of MI in Q5 in our baseline data. In this context, higher SHR values predominantly serve as a reflection of CAD severity rather than a causative factor. Furthermore, our findings suggest that while higher SHR values reflect CAD severity, lower SHR values may contribute to increased CAD complexity as an etiological factor. This is evidenced by our observation of a greater proportion of diabetic patients with elevated HbA1c levels and lower acute blood glucose levels in both Q1 and Q5 of our baseline data. An SHR < 0.68 indicates a combination of chronic hyperglycemia (high HbA1c) and currently lower blood glucose (low acute blood glucose). Previous studies have extensively established chronic hyperglycemia as a risk factor for CAD severity [12, 13, 15, 33, 34], and additional studies have shown that oscillating glucose can have more detrimental effects than constant high glucose on endothelial function and oxidative stress, suggesting that recurrent low acute blood glucose also contributes to CAD progression [42, 43]. Therefore, in all scenarios where the linear correlation between ABG and HbA1c deviates, the OR of the mid/high SYNTAX score increases through different mechanisms. ACS is an acute condition that requires prompt assessment and treatment planning. The U-shaped relationship between the SHR and SYNTAX can facilitate rapid and efficient assessment of disease conditions. However, there is limited research in this area, with inconsistent diagnostic thresholds. Our study also differs from previous short- and long-term prognosis studies in terms of thresholds. Future research should focus on larger-scale prospective cohort studies to determine diagnostic thresholds for the SHR and explore its predictive value for CAD complexity in ACS patients.

Furthermore, subgroup analyses generally aligned with overall population trends, except for the subgroup with DM, where their interaction exhibited a marginally significant difference (P for interaction = 0.045). Specifically, the U-shaped associations of the SHR with the mid/high SYNTAX score were attenuated in the DM subgroup. This difference primarily occurred within the SHR < 0.68 interval, where the OR trend in the DM group was weaker than that in the non-DM group, while within the SHR > 0.875 interval, both groups showed consistent OR trends. This phenomenon is somewhat consistent with the findings of a previous study. Zhang Y et al. reported that SHR (as both a continuous variable and categorized into 3 tertiles) was significantly correlated with an increased risk of MVD in the pre-DM and DM groups, but no consistent linear increase was observed in the normal glucose regulation (NGR) group [20]. However, this study seemingly did not further explore its potential nonlinear trends. Additionally, several studies have demonstrated a substantial correlation between HbA1c levels and the SYNTAX score, even among individuals without DM [12, 13, 15, 33, 34]. For example, Garg N et al. found that the HbA1c level had a linear incremental association with CAD in 1141 nondiabetic individuals, and the HbA1c level was also independently correlated with disease severity and higher SYNTAX scores. These findings could explain the U-shaped relationship observed in the non-DM subgroup in the present study. Moreover, Yan Y et al. suggested that the positive association between HbA1c and the SYNTAX score might be notably attenuated by prior medication history when included in the adjusted model [14]. Given the absence of detailed medication history data (particularly regarding antidiabetic therapy) in our study, disparities among DM and non-DM subgroups may result from unadjusted factors such as prehospital medication therapy or limitations in sample size. In other subgroups, no significant interaction was found, including the sex subgroup of particular interest. The shape of the curves for both sexes is consistent with the overall curve (Figure S1). Although the P for nonlinearity is 0.004 for the male subgroup and 0.521 for the female subgroup, interaction analysis suggests no significant interaction by sex (P for interaction = 0.732). We believe that the differences observed between the sex subgroups may be due to the smaller sample size.

### Strengths and limitations

This study is the first to investigate the role of the SHR in assessing CAD complexity and severity using the SYNTAX score in patients with ACS. Furthermore, for the first time, we explored the nonlinear relationship between the SHR and CAD complexity, suggesting a U-shaped association between the SHR and mid/high SYNTAX score in ACS patients. However, there are limitations to our study. First, this study is based on an Asian cohort, and the extrapolation of its conclusions to other racial groups requires further validation. Second, the data were obtained from a PCI cohort, which excluded patients with low CAD complexity not requiring revascularization and those with high CAD complexity undergoing CABG, potentially introducing bias. Future research should validate our results in more comprehensive CAG cohorts. Third, we cannot completely rule out the possibility of unrecorded or unknown confounding factors, such as prehospital medication history, which may influence the associations observed in this study. Furthermore, this study discovered distinct distribution characteristics, such as gender factors in the variation of SHR. Further research can delve into this aspect to explore more directions in CAD studies.

## Conclusions

There were U-shaped associations between the SHR and CAD complexity, as assessed by the SYNTAX score, in ACS patients, with an SHR ranging from 0.68 to 0.875 indicating a relatively lower OR for mid/high SYNTAX scores. Further studies are necessary to both evaluate the predictive value of the SHR in ACS patients and explore the underlying mechanisms of the observed U-shaped associations.

### Electronic supplementary material

Below is the link to the electronic supplementary material.

Supplementary Material 1: Additional file 1: Table S1. The variance inflation factor (VIF) and tolerance of covariates; Additional file 1: Figure S1. Restricted cubic splines for the odds ratio of mid/high SYNTAX score in males (A) and females (B). Adjusted for age, body mass index, current smoker, DM, hypertension, dyslipidemia, previous MI, previous stroke, PVD, eGFR, TG, HDL-C, LDL-C, Lp(a), hs-CRP, uric acid, LVEF < 40%, and clinical presentation. Abbreviations as shown in Table 1 .

## Data Availability

The datasets used in the study are available from the corresponding author upon reasonable request.

## Abbreviations

ABG: blood glucose
ALB: albumin
BMI: body mass index
COPD: chronic obstructive pulmonary disease
CTO: chronic total occlusion
DBP: diastolic blood pressure
eGFR: estimated glomerular filtration rate
Hs-CRP: high-sensitivity C-reactive protein
HDL-C: high-density lipoprotein
LDL-C: low-density lipoprotein
LM: left main disease
LVEF: left ventricular ejection fraction
MI: myocardial infarction
MVD: multivessel disease
NSTEMI: non-ST-segment elevation myocardial infarction
PVD: peripheral vascular disease
SBP: systolic blood pressure
STEMI: ST-segment elevation myocardial infarction
TG: triglyceride
TC: total cholesterol
UA: unstable angina
SYNTAX: Synergy between PCI with TAXUS™ and Cardiac Surgery
