# Supplementary material for: Association of the stress hyperglycemia ratio with coronary artery disease complexity as assessed by the SYNTAX score in patients with acute coronary syndrome
Source: Diabetol Metab Syndr. 2024 Jun 25;16:139. doi: 10.1186/s13098-024-01382-0 (PMC11197361; doi:10.1186/s13098-024-01382-0)
Supplement: Supplementary file 1 — Supplementary Material 1: Additional file 1: Table S1. The variance inflation factor (VIF) and tolerance of covariates; Additional file 1: Figure S1. Restricted cubic splines for the odds ratio of mid/high SYNTAX score in males (A) and females (B). Adjusted for age, body mass index, current smoker, DM, hypertension, dyslipidemia, previous MI, previous stroke, PVD, eGFR, TG, HDL-C, LDL-C, Lp(a), hs-CRP, uric acid, LVEF < 40%, and clinical presentation. Abbreviations as shown in Table 1 . [file 13098_2024_1382_MOESM1_ESM.docx]

Table S1. The variance inflation factor (VIF) and tolerance of covariates.

| Variable | VIF | Tolerance |
| --- | --- | --- |
| Crude | 1.097 | 0.911577 |
| Age, year | 1.961 | 0.509944 |
| Male, n (%) | 1.628 | 0.614251 |
| BMI, kg/m^2^ | 1.133 | 0.882613 |
| Current smoker, n (%) | 1.405 | 0.711744 |
| Hypertension, n (%) | 1.131 | 0.884173 |
| Dyslipidemia, n (%) | 1.062 | 0.94162 |
| Previous MI, n (%) | 1.911 | 0.523286 |
| Previous stroke, n (%) | 1.184 | 0.844595 |
| PVD, n (%) | 1.3 | 0.769231 |
| eGFR, mL/min/1.73m^2^ | 1.109 | 0.901713 |
| LVEF <40%, n (%) | 1.059 | 0.944287 |
| Clinical presentation | 1.229 | 0.81367 |
| TG, mmol/L | 1.362 | 0.734214 |
| HDL-C, mmol/L | 1.036 | 0.965251 |
| LDL-C, mmol/L | 2.805 | 0.356506 |
| Lp (a), mg/L | 1.078 | 0.927644 |
| Hs-CRP, mg/L | 1.031 | 0.969932 |
| Uric acid, mmol/L | 2.655 | 0.376648 |

Abbreviations: BMI body mass index, eGFR estimated glomerular filtration rate, Hs-CRP high-sensitivity C-reactive protein, HDL-C high density lipoprotein, LDL-C low density lipoprotein, LVEF left ventricular ejection fraction, MI myocardial infarction, PVD peripheral vascular disease, TG triglyceride.


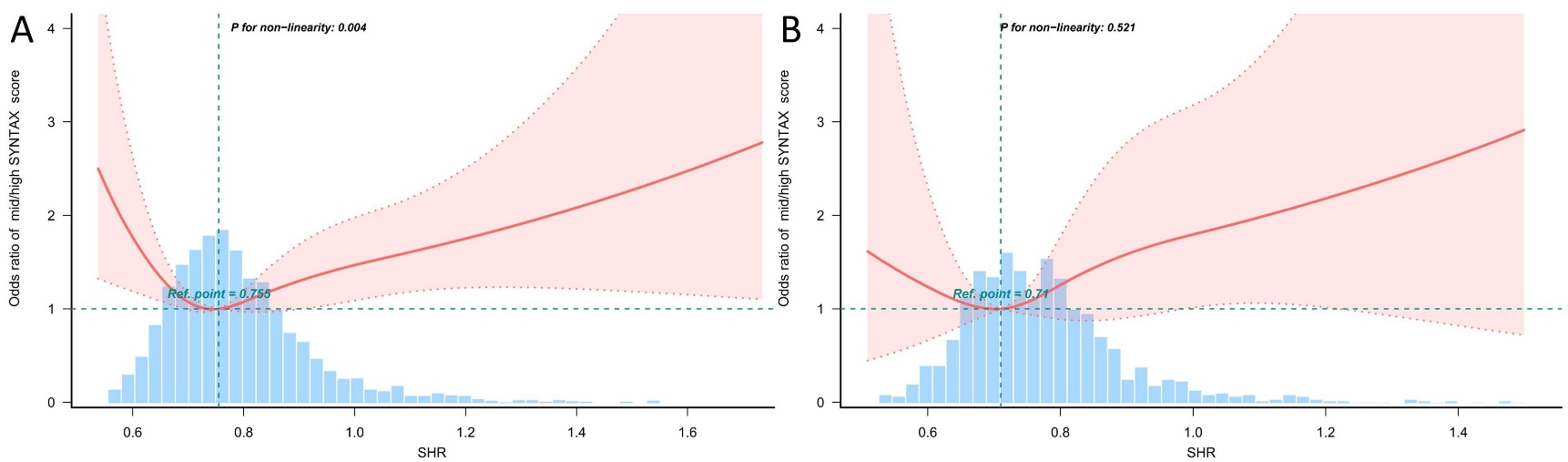


**Figure S1. Figure S1. Restricted cubic splines for the odds ratio of mid/high SYNTAX score in males (A) and females (B).** Adjusted for age, body mass index, current smoker, DM, hypertension, dyslipidemia, previous MI, previous stroke, PVD, eGFR, TG, HDL-C, LDL-C, Lp(a), hs-CRP, uric acid, LVEF < 40%, and clinical presentation. Abbreviations as shown in Table 1.
